# How does mitochondria function contribute to aerobic performance enhancement in lizards?

**DOI:** 10.3389/fphys.2023.1165313

**Published:** 2023-05-05

**Authors:** Kara M. Reardon, Brenna N. Walton, Jerry F. Husak

**Affiliations:** Department of Biology, University of St. Thomas, Saint Paul, MN, United States

**Keywords:** aerobic exercise, endurance, metabolism, mitochondria, lizard

## Abstract

**Aims:** Aerobic exercise typically enhances endurance across vertebrates so that chronically high energy demands can be met. Some known mechanisms of doing this include increases in red blood cell numbers, angiogenesis, muscle fiber adaptions, mitochondria biogenesis, and changes to cellular metabolism and oxidative phosphorylation. We used green anole lizards (*Anolis carolinensis*) to test for an effect of aerobic exercise on metabolism, mitochondria densities, and mitochondrial function.

**Methods:** We first tested the response of green anoles to endurance training and pyrroloquinoline quinone (PQQ) supplementation, which has been shown to increase mitochondria biogenesis. We also conducted a mitochondrial stress test to determine how training affected mitochondrial function in skeletal muscle fibers.

**Results:** Aerobic exercise led to increased endurance and decreased standard metabolic rate (SMR), while PQQ did not affect endurance and increased SMR. In a second experiment, aerobic exercise increased endurance and decreased resting metabolic rate (RMR) in both male and female green anoles. Higher counts of mitochondrial gene copies in trained lizards suggested additional mitochondria adaptations to achieve increased endurance and decreased metabolism. A mitochondrial stress test revealed no effect on baseline oxygen consumption rates of muscle fibers, but untrained lizards had higher maximal oxygen consumption rates with the addition of metabolic fuel.

**Conclusion:** It is likely that trained lizards exhibited lower maximal oxygen consumption rates by developing higher mitochondria efficiency. This adaptation allows for high ATP demand to be met by making more ATP per oxygen molecule consumed. On the other hand, it is possible that untrained lizards prioritized limiting reactive oxygen species (ROS) production at rest, while sacrificing higher levels of proton leak and higher oxygen consumption rates when working to meet high ATP demand.

## 1 Introduction

Mitochondria synthesize ATP, which is required for a multitudie of bodily functions that are important for Darwinian fitness, including reproduction, immunity, and locomotion. However, mitochondria are not static organelles, and mitochondrial adaptations allow organisms to respond to a variety of stressors and changing energy demands via plastic responses, such as increasing mitochondrial density or respiratory capacity and minimizing oxidative stress, among others (Holloszy & Coyle, 1984; Yan et al., 2011; Conley, 2016; Hwang and Willoughby, 2018; Heine & Hood, 2020). Although, the complexity of mitochondrial adaptations is not yet fully understood, it is evident that the ability to change mitochondrial properties in response to changing energy demands is critical to survival through maintaining aerobic capacity (Blackstone and Chang, 2011; Hood et al., 2018). Plasticity in mitochondrial function allows animals to maintain and optimize whole organism performance across challenging environments such as intertidal zones, high altitudes, or thermal extremes (Salin et al., 2015; Hood et al., 2018) and has been observed across a diverse range of species. For example, oysters tolerate hypoxic conditions in intertidal zones through mitochondrial proteome shifts important for upregulating electron transport, limiting electron flow, and maintaining energy-saving homeostasis (Sokolov et al., 2019). Honeybees deal with extreme temperature changes through mitochondria resiliency, increased oxygen consumption, and substrate oxidation flexibility (Hichem et al., 2022). Although mitochondria densities differ among tissues within an individual organism, and among the same tissues across organisms, it is less understood how mitochondrial adaptations within various *in vivo* tissues affect whole organism performance and aerobic capacity, especially under changing energy demands (Monaghan et al., 2009; Hood et al., 2018; Koch et al., 2021).

Chronic increased aerobic activity can lead to rapid performance enhancement. The classic example of this is the exercise response in humans, which results in numerous coordinated physiological changes in multiple body systems (Holloszy & Coyle, 1984; Bloise, Cordeiro, & Ortiga-Carvalho, 2018). Similarly, ectothermic lizards increase heart size and red blood cell counts in response to endurance-exercise training (Husak et al., 2015; 2016). However, these cardiovascular changes alone probably do not explain the dramatic increase in aerobic performance (Husak et al., 2015), and mitochondrial changes are likely important. In addition to increased mitochondria densities in response to aerobic exercise (Heine & Hood, 2020), studies of mammals show that increased activity can also result in functional mitochondrial adaptations, such as changes in substrate oxidation, affinity of enzymes for oxygen and ADP, and biochemical coupling efficiency within mitochondria of skeletal muscle cells (Yan et al., 2011; Boushel et al., 2014; Conley, 2016). Additionally, mitochondria have been found to increase inner mitochondrial membrane densities and inter-mitochondrial junctions, as well as fission and fusion of whole mitochondria, all of which can alter mitochondria function and efficiency (Heine & Hood, 2020). Despite these benefits as a response to increased activity, aerobic exercise also requires immediate energy investment and has been shown to result in both short- and long-term costs in the form of phenotypic tradeoffs (Husak et al., 2016; 2017; 2021). However, the mechanisms that enhance endurance while also producing widespread phenotypic tradeoffs remain poorly understood.

Aerobic exercise leads to increased energy demand, and one way organisms meet this demand is to upregulate the expression of the peroxisome proliferator-activated receptor gamma (PPAR-γ) gene, which increases mitochondria biogenesis (Wright et al., 2006). There is evidence that mammals do this by regulating thyroid hormones to promote muscle fiber adaptation which increases the expression of part of the PPAR-γ gene, the peroxisome proliferator-activated receptor-gamma coactivator (PGC-1α) (Bloise et al., 2018). PGC-1α is a key transcription factor for skeletal muscle fiber type composition, fatty acid oxidation, and particularly for promoting mitochondria biogenesis and oxidative metabolism in metabolically active tissues (Liang & Ward, 2006). PGC-1α also plays a key role in regulating oxidative phosphorylation, reducing toxic reactive oxygen species (ROS) and mitochondrial toxins, and breaking down lipid and carbohydrate energy stores (Liang & Ward, 2006; Wright et al., 2006; Leick et al., 2008; Rucker et al., 2009). Because of these effects, PGC-1α is important for enhancing endurance performance in mammals, but it is unknown if mitochondria synthesis, regulated by PGC-1α, contributes significantly to enhanced endurance performance in non-mammals.

We sought to determine what physiological changes occur in green anole lizards (*Anolis carolinensis*) in response to aerobic exercise training to improve endurance performance. Previous work showed that laboratory-trained green anoles enhance their endurance performance over two-fold in response to aerobic exercise training (Husak et al., 2015; 2016), but endurance training also lowers standard metabolic rate (SMR; Lailvaux et al., 2018) If mitochondria biogenesis alone explained the performance enhancement, then SMR, all else equal, should have been increased instead of decreased after training (Maurya et al., 2018). Thus, there must be other mechanisms to account for the observed magnitude of enhanced endurance performance, including changes to mitochondrial function. We thus completed a series of experiments to explore several possibilities of how mitochondria might be involved in endurance-performance enhancement via aerobic exercise. In our first study, to understand how mitochondrial number impacts endurance, we examined the effect of endurance training and the supplement pyrroloquinoline quinone (PQQ) on aerobic performance, SMR, and mitochondrial gene copy number. PQQ can increase mitochondria biogenesis by increasing the expression of PGC-1α (Rucker et al., 2009; Jonscher et al., 2021). PQQ stimulates 5’ AMP-activated protein kinase (AMPK) which activates cAMP response element binding protein (CREB). CREB and PGC-1α bind to DNA sequences that regulate mitochondria biogenesis. Nuclear respiratory factor 1 (NRF-1) is also stimulated by PQQ and works as a transcription factor with CREB to increase the expression of necessary mitochondrial subunits. We predicted an increase in mitochondria densities in both endurance trained and PQQ-supplemented lizards due to presumed increased expression of PGC-1α. Additionally, the combination of training and PQQ may have the highest impact on endurance if the mechanism of change is similar for the two treatments; that is, there may be additive effects on performance. In our second study, we predicted adaptations to mitochondrial function in the skeletal muscle of endurance-trained lizards compared to controls. We expected endurance training to affect basal mitochondrial respiration, non-mitochondrial respiration, ATP synthase function, proton leak, maximal respiration, and spare respiratory capacity. This combination of studies should shed light on how ectothermic lizards increase endurance capacity when energetically challenged with increased activity in the form of aerobic exercise training.

## 2 Materials and methods

### 2.1 General husbandry

Adult male and female green anoles (*Anolis carolinensis*), were obtained (Candy’s Quality Reptiles, Laplace, LA, USA) and housed in a climate-controlled room for 2 weeks of acclimation at 28–31°C on a 12:12 h light:dark cycle. Each cage had either a single male or a male-female pair (see below) so that reproduction and reproductive behavior would be maintained where appropriate. Lizards were each fed four crickets 3 days a week, and crickets were dusted with calcium and vitamin D supplements on one of those days (Husak et al., 2016; Husak et al., 2017; Wang, 2020). Cages were sprayed twice daily to provide water. Prior to all experiments, we measured snout-vent length (SVL), body mass, and endurance capacity (described below) for all lizards. All procedures were in accordance with the University of St. Thomas Institutional Animal Care and Use Committee.

### 2.2 Effects of PQQ supplementation and training

The first study group included 44 adult male lizards, 12 given PQQ supplement, 9 trained, 10 given PQQ supplement and trained, and 13 left untreated.

#### 2.2.1 Endurance training and PQQ supplement treatments

The green anole lizards assigned to the training treatment were aerobically exercised on treadmills moving 0.18 km/hr three times a week for 6 weeks to improve their endurance. We increased the training time by 10 min per session every 2 weeks beginning with 25 min per session for the first 2 weeks and ending with 45 min per session for the last 2 weeks. This exercise regime was designed to mimic the upper bounds of natural activity in nature, and thus represents a biologically relevant manipulation (Husak et al., 2015). Our training procedures were designed to represent a realistic simulation of high-end performance by green anoles as we describe in more detail elsewhere (Wang, 2020). For example, a 30-min training session equals 90 m of slow traveling by a lizard, which is ecologically relevant to green anoles in nature (Irschick, 2000), where –25% of their time is spent traveling through their home ranges, and much of this time is “creeping” at lower speeds (Jenssen et al., 1995). Green anoles use slow, sustained locomotion for territory patrolling and foraging, and our training regime represented performance at the high end of distance traveled in a day and speeds used in nature; that was precisely our objective—to determine how high levels of performance use might impact mitochondria in skeletal muscle.

Throughout the 6 weeks of the experiment, the PQQ-supplemented lizards were given 0.002 mM (Stites et al., 2006) of PQQ three times a week by dissolving PQQ disodium salt in water and injecting the solution into crickets which were then fed to the lizards.

We measured the final endurance of all the lizards by running them on treadmills at a standard speed of 0.3 km/hr and recording the time at which each reached the point of exhaustion, which is when the lizard could no longer right itself after being flipped on its back (Perry et al., 2004; Cox et al., 2009; Husak et al., 2015).

#### 2.2.2 Standard metabolic rate (SMR)

After 6 weeks of training, between 2,100 and 0400 h, we followed the standard respirometry procedures outlined in Lailvaux et al. (2018) to measure SMR. We placed each lizard in an incubator at 28°C (Orrell et al., 2004) inside a plastic 60-mL syringe connected to a Qubit flow-through respirometry system that measured the flux in carbon dioxide concentrations over thirty minutes for each lizard after allowing their rates to stabilize from any handling-induced stress. Lizards were fasted starting the day before trials were conducted.

#### 2.2.3 Mitochondrial gene copy number

We quantified mitochondrial gene copy number as a proxy for the number of mitochondria to test for mitochondrial biogenesis. Although mtDNA copy number does not always correlate well with measures of mitochondrial function (Larsen et al., 2012), it is reasonable to estimate mitochondrial biogenesis by determining the number of mitochondrial genomes per unit weight of tissue (Longchamps et al., 2020). After completion of data collection, the anoles were euthanized, and the gastrocnemius muscle was rapidly extracted. Each muscle was weighed, flash frozen, and stored at −20**°**C. Genomic DNA (gDNA) was isolated from each muscle sample (approximately 5 mg) using Qiagen DNeasy Plant Mini Kits (Qiagen, Hilden, Germany), with the addition of a 1h incubation in lysis buffer supplemented with 20 mg/mL proteinase K at 65**°**C. Samples were vortexed every 10 min to promote tissue lysis. DNA was quantified on a Nanodrop 2000 (ThermoFisher Scientific, Waltham, Massachusetts) and stored at −20**°**C. PCR products from each target gene were amplified, cloned into the pGemT-EZ vector (Promega, Madison, WI), and used to transform DH10B cells. Transformants were selected on ampicillin media and purified plasmid DNA was sequenced to confirm the identity of each clone. Isolated plasmid was digested with XmnI to linearize the plasmids, and standard curves were created using serial dilutions of digested plasmids. Plasmid dilutions containing 300-300,000 copies of the cloned target gene were adjusted to have the same total DNA concentration (20 ng/uL) using calf thymus DNA. This was done to simulate the amplification conditions present in total genomic DNA isolated from anole tissue.

Mitochondrial DNA copy number was calculated relative to the single-copy nuclear gene HSD17. Primers for two mitochondrial targets, mtA and mtB (different sections of NADH dehydrogenase subunit 2[ND2]), and HSD17 were designed (Liu et al., 2017), and ordered from Integrated DNA Technologies, Coralville, Iowa (see Supplementary Table S1). The primers for HSD17 and mtB had an annealing temperature of 52.3**°**C, while mtA primers had an annealing temperature of 59**°**C.

Before use in quantitative PCR (qPCR), each DNA sample was diluted to 3.125 ng/uL in Qiagen AE buffer. If additional dilution was necessary due to early amplification, samples were further diluted in calf thymus DNA. All qPCR reactions used the Promega Go-Taq qPCR system (Madison, Wisconsin). A total of 2 uL of sample DNA was added to each qPCR plate well to yield a total loading volume of 25 uL. Samples were loaded in triplicate. No template controls were also utilized, where 2 uL of nuclease free water was loaded in triplicate instead of sample DNA. Each qPCR run was programmed on a StepOnePlus thermal cycler (ThermoFisher Scientific, Applied Biosystems, Coralville, Iowa) to heat the sample at 95**°**C for 10 min, then run 40 cycles of 95**°**C for 10 s, 52.3**°**C or 59**°**C for 30 s, and 72**°**C for 30 s. A melt curve was included at the end of the run to ensure the amplified product was specific to the intended target (Christoffolete et al., 2006).

The cycle threshold (CT) for each sample triplicate was averaged to get an accurate cycle amplification. The standard curve was utilized to calculate mitochondrial copy number (CN) from the CT according to Miller et al., 2003. A ratio of mitochondrial target CN to the nuclear target CN was then calculated.

#### 2.2.4 Data analysis

Change in endurance data were analyzed using a two-way analysis of variance (ANOVA) by using the difference between the final endurance and the baseline endurance against the treatments of training and supplementing. SMR data were log10 transformed and analyzed using a two-way analysis of variance. Preliminary analyzes showed that SMR did not scale to body mass in this group with a narrow range of masses (*p* = 0.65), so mass was not included as a covariate. We analyzed mitochondrial-to-nuclear CN ratio using two-way ANOVA, with training treatment and supplement treatment as factors. Analyses were performed in JMP 14 (SAS Institute, Cary, NC).

### 2.3 Mitochondria function—mitochondrial stress test

#### 2.3.1 Endurance training and metabolic measurements

The second experiment included 19 adult male and 19 adult female lizards, with 9 of each sex endurance trained and 10 of each sex used as sedentary controls. A similar training procedure was used as in the first experiment, with lizards aerobically exercised three times a week on treadmills for 6 weeks. We increased the training time by 10 min per session every 2 weeks to build endurance. For this experiment we began with 20 min per session for the first 2 weeks and ended with 40 min per session in the last 2 weeks. The slightly different times of training were due to logistical constraints and had no apparent impact on the experiment. We measured whole-organism metabolic rates as described for the first experiment above, except that we measured them during the day instead of at night. Because this violates one of the assumptions of what defines an SMR, we refer to measures in this second experiment, by convention, as resting metabolic rates (RMR). Previous work in our laboratory has shown that SMR and RMR are correlated and respond to training similarly (Lailvaux et al., 2018).

The procedure used to dissociate the gastrocnemius muscle was modified from similar protocol for measuring mitochondria respiration in intact skeletal muscle fibers with a Seahorse XF24/XFe24 Analyzer (Agilent Technologies, 2016). Previous studies have measured mitochondrial respiration through methods such as permeabilizing muscle fibers or isolating mitochondria. However, by maintaining intact muscle cells, we were able to analyze mitochondria function within ecologically relevant connected cellular networks (Koch et al., 2021). The XF Cell Mito Stress Test (Agilent Technologies, 2016) measures maximal oxygen consumption rates, but our modification controlled for substrate availability with the addition of pyruvate, ensuring that the maximal respiration measurements in muscle cells were not substrate limited. The right gastrocnemius muscle was extracted from each freshly euthanized lizard, rinsed in PBS and incubated for 20 h overnight in dissociation medium, a high glucose solution containing gentamycin and collagenase with pH 7.2 (Agilent Technologies, 2016). The following morning, two Seahorse sensor cartridges were hydrated in Seahorse XF Calibrant for a minimum of four hours. The muscle fibers were transferred from the dissociation medium to the incubation medium with a small bore (∼1 mm) fire polished glass transfer pipette by gently sucking the muscle into the tip of the pipette. A 3 mL plastic transfer pipette was used to triturate the muscles and isolate single muscle fibers. Once the muscle fibers were separated, debris and nerve tissue were removed with forceps.

Growth Factor Reduced BD Matrigel Matrix (Corning Life Sciences, 354230) was diluted 1:1 with DMEM and 5 μL was added to each well of the Agilent Seahorse XFe24 Cell Culture Microplates. The plate was tapped vigorously against the hand to allow the Matrigel to cover the entirety of each well. Once the plate had air dried for 20 min, 90 μL aliquots of the isolated muscle fibers were transferred to the cell culture microplates by targeting the most intact muscle fibers and avoiding debris. We ensured muscle fibers covered 50%–60% of the bottom of the well. Although we did not normalize cellular metabolism to protein content, we are confident that our measures across treatments are comparable. We visually confirmed equal isolation efficiency of muscle fibers, and our results are the opposite of what one would predict if trained lizards had more protein (and higher oxygen consumption) compared to controls. The muscle fibers were left for 1 h in a 28°C incubator to attach. We aspirated 40 μL of the incubation medium from each well, added 300 μL of assay medium and then removed it from each, and then added a final 450 μL of assay medium to each well for a total of 500 μL. Background correction wells had 500 μL of assay medium added.

We measured oxygen consumption rate (OCR) of muscle fibers following modified protocols for the Agilent Seahorse XF Cell Mito Stress Test (Agilent Technologies, 2016). In brief, OCR was measured at baseline and then after a series of reagents were automatically added to wells by the Seahorse. First, 1 μM of oligomycin was added, which inhibits complex V of ATP synthase. Then, 0.4 μM of FCCP was added, which collapses the proton gradient by uncoupling the electron transport chain from ATP synthase. Next, 10 μM of pyruvate was added, which ensures enough NADH can be made to push electrons through the, ETC. Finally, 0.5 μM of rotenone/antimycin was added to inhibit complexes I and III. OCR measurements were recorded every 10 min (3x for each of these 5 steps). From these measurements, we were able to examine differences in OCR due to sex, whether trained or not, and mito stress treatment. Although we should have been able to calculate mitochondrial proton leak by subtracting OCR after rotenone/antimycin from OCR after oligomycin, we were unable to get OCR low enough after the addition of rotenone/antimycin. Therefore, we focused on evaluating differences for the other treatments (baseline, oligomycin, FCCP, pyruvate). By examining where differences existed during the mito stress test we could make inferences about differences due to training for basal respiration (OCR prior to manipulation), ATP linked respiration (difference in OCR before and after oligomycin), and maximal respiration (after addition of FCCP and pyruvate) before and after pyruvate. A significant interaction between training and mito stress treatment would indicate that training affected OCR differently at some or all the stages of the mito stress test.

#### 2.3.2 Data analysis

We tested for differences in endurance and RMR using two-way ANCOVA, with sex and training used as factors, and body mass as a covariate. We used R version 3.6.0 (R Core Team 2019) to examine changes in OCR due to sex, training, and mito stress test treatments. To test the hypothesis that training and mito stress test manipulations affect OCR, we fit general linear mixed-models to OCR using the R package *lme4* (Bates et al., 2015). Fixed factors in all cases were sex, training treatment, mito stress treatment, and the training*mito stress treatment interaction, whereas individual ID was included as a random effect to account for the repeated measurements across the Mito Stress Test.

## 3 Results

### 3.1 Effects of PQQ supplementation and training

**FIGURE 1:**
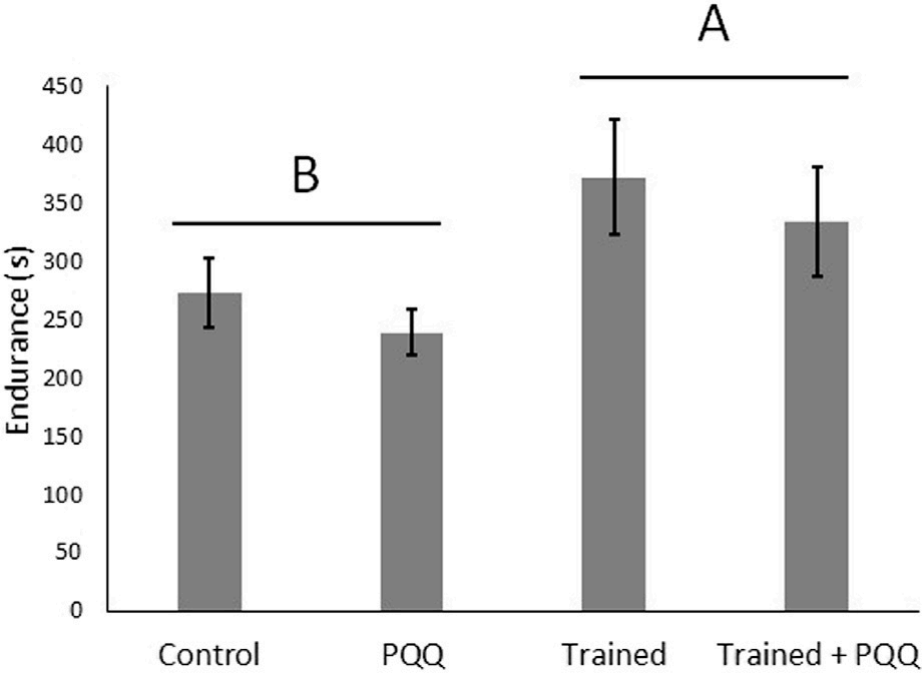
Aerobic exercise increases endurance but PQQ supplementation does not. We analyzed change in endurance but show final, post-training endurance for illustrative purposes. Different letters over different groups of bars represent a significant effect of training.

**FIGURE 2:**
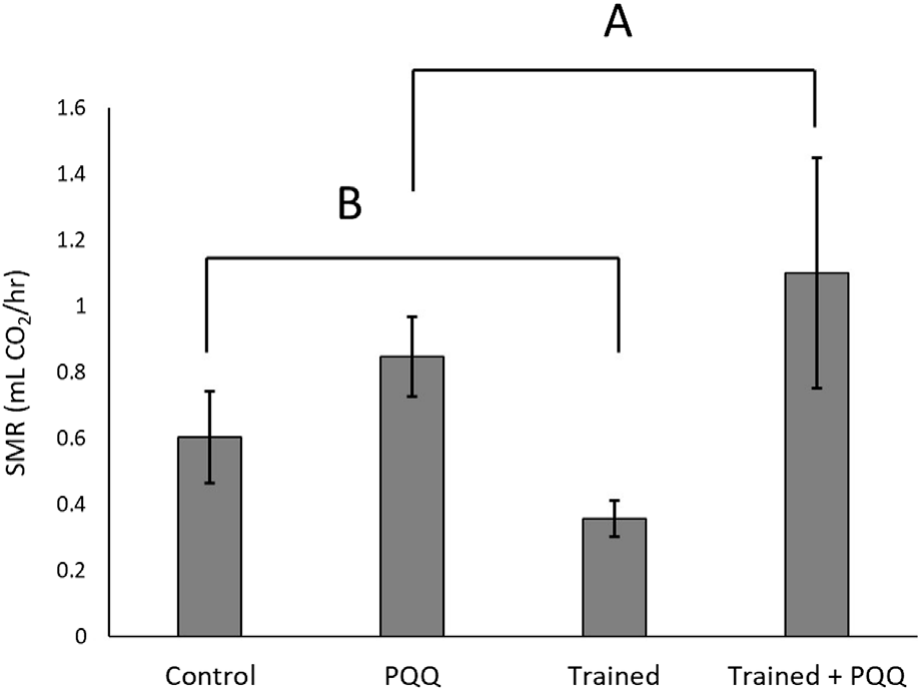
Standard metabolic rates increased with PQQ supplement. There was no significant effect of training on SMR, but we note that trained-only lizards had the lowest SMRs. Different letters over different groups connected by brackets represent a significant effect of PQQ supplementation.

The first experiment consisted of male lizards only with training and PQQ supplementation. There was a significant effect of training on change in endurance (F_1,40_ = 9.66, *p* = 0.004; Figure 1), but there was no significant effect of PQQ supplement on change in endurance (F_1,40_ = 0.69, *p* = 0.41), nor was there a significant training*supplement interaction (F_1,40_ = 0.003, *p* = 0.96). There was no significant effect of training on SMR (F_1,40_ = 0.13, *p* = 0.72), but lizards with PQQ supplements had significantly higher SMRs compared to controls (F_1,40_ = 15.64, *p* = 0.0003; Figure 2). The training*supplement interaction was not significant (F_1,40_ = 1.41, *p* = 0.24). However, we note that trained-only lizards had the lowest SMRs, almost four-fold less than the trained + PQQ group (Figure 2), which matches our previous work (Lailvaux et al., 2018).

**FIGURE 3:**
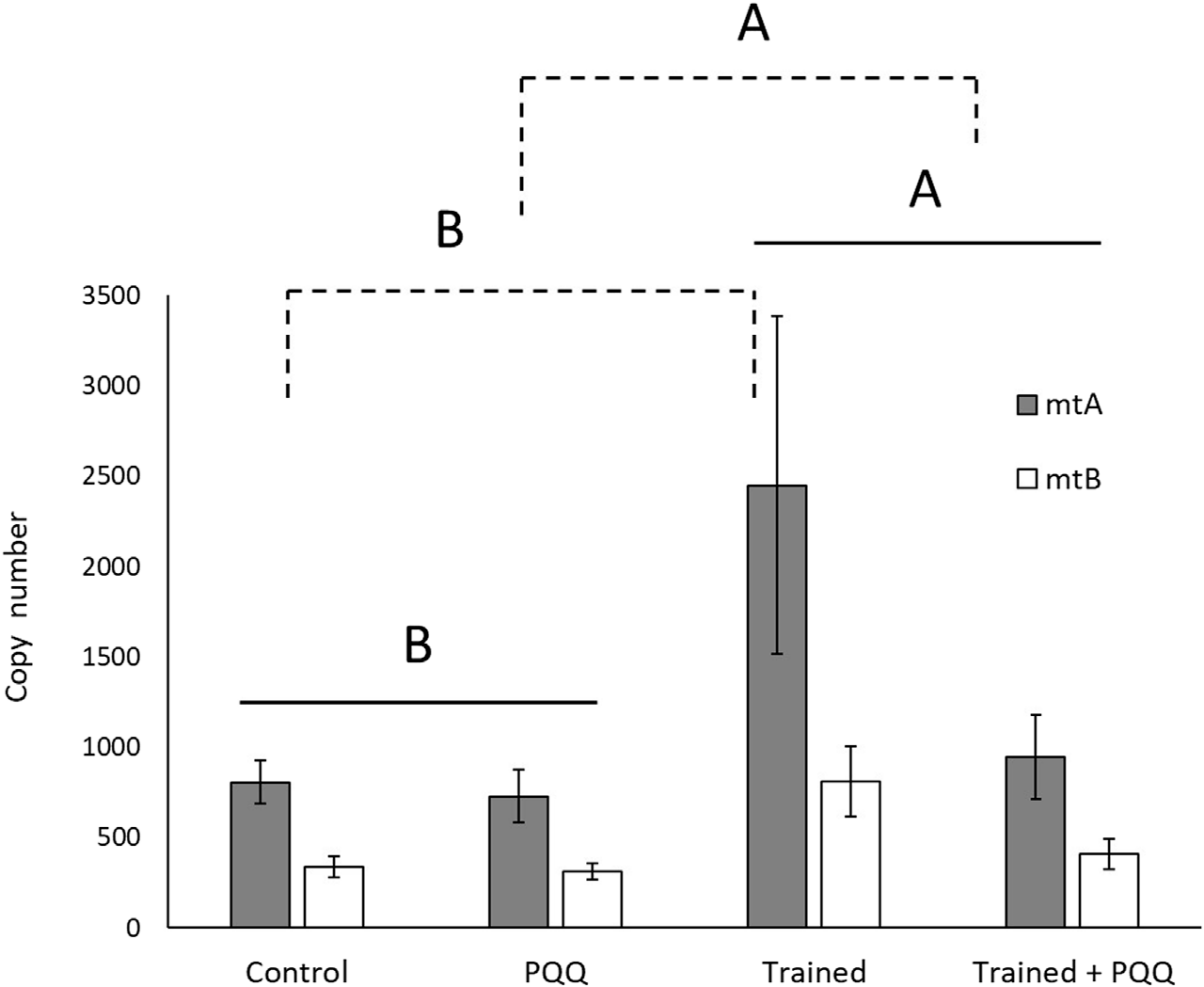
Mitochondrial DNA copy number in gastrocnemius muscle increased with aerobic exercise training (shown with solid lines) but decreased with PQQ supplements (shown with dashed lines). Different letters over different groups of bars or groups connected with brackets indicate a significant difference.

There was no significant training*supplement interaction for mitochondrial copy number (mtA, F_1,24_ = 2.70, *p* = 0.11; mtB, F_1,26_ = 3.68, *p* = 0.07). There was a significant effect of training for both copy number targets (mtA, F_1,24_ = 4.61, *p* = 0.04; mtB, F_1,26_ = 8.68, *p* = 0.007; Figure 3), with trained lizards having a greater copy number. There was a significant effect of PQQ supplement for target mtB (F_1,26_ = 4.73, *p* = 0.04), and a difference approaching significance for mtA (F_1,24_ = 3.31, *p* = 0.08), with PQQ-supplemented lizards having lower copy numbers compared to saline controls (Figure 3).

### 3.2 Mitochondria function—Mito stress test

**FIGURE 4:**
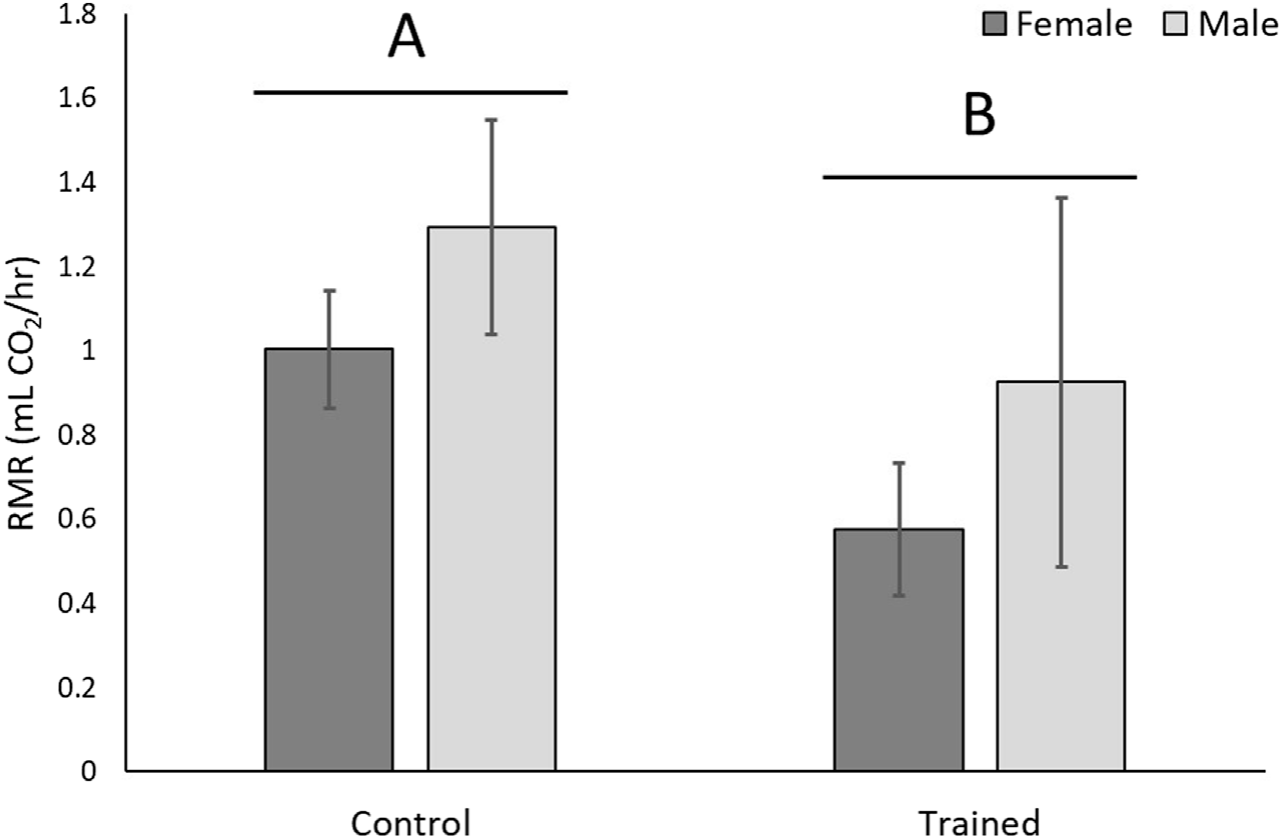
Whole-organism resting metabolic rate (RMR) decreased with aerobic exercise training. Absolute metabolic rates are shown, but when corrected for body size females had significantly higher RMRs than males. Different letters over different groups of bars represent a significant effect of training.

In the second experiment, male and female lizards were endurance trained. There was a significant effect of training on endurance (F_1,31_ = 17.20, *p* = 0.0002), but there was no significant effect of sex on endurance (F_1,31_ = 2.26, *p* = 0.14), nor was there a significant training* sex interaction (F_1,31_ = 1.21, *p* = 0.28). Trained lizards had lower RMRs than controls (F_1,31_ = 4.02, *p* = 0.05; Figure 4), and females had higher RMRs than males (F_1,31_ = 19.38, *p* = 0.0001), but there was no significant training*sex interaction (F_1,31_ = 0.53, *p* = 0.47). Figure 4 shows mean (±SEM) of absolute RMRs, but correction for body size with ANCOVA gave least square means of 1.66 ± 0.18 mL CO_2_/hr for females and 0.21 ± 0.18 mL CO_2_/hr for males.

**FIGURE 5:**
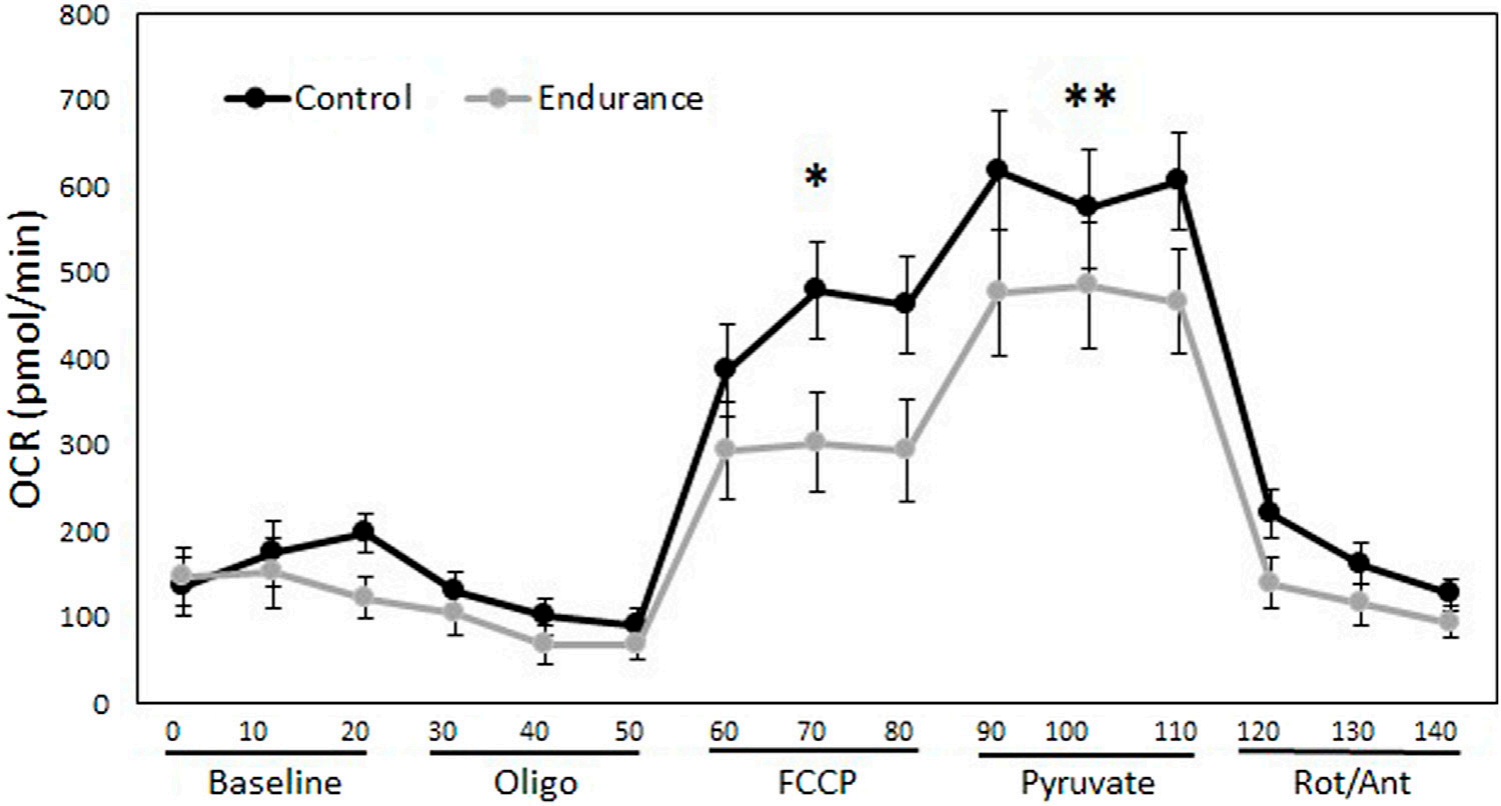
Aerobic exercise alters mitochondrial function but not baseline oxygen consumption of muscle fibers. This figure demonstrates the effect of endurance training on oxygen consumption rates (OCR) of gastrocnemius muscle fibers over 140 min. The addition of reagents allows changes to be seen in mitochondrial basal respiration, ATP linked respiration, and maximal respiratory capacity. Trained lizards did not experience as much of an increase in OCR as control lizards after the addition of FCCP and pyruvate, indicating lower maximal respiratory capacity. There was no difference between trained and control lizards OCR at baseline. Oligo = oligomycin, FCCP = carbonyl cyanide-*p*-trifluoromethoxyphenylhydrazone, Rot/Ant = rotenone and antimycin. *p < 0.05, **p < 0.01.

There was a significant training*mito stress treatment interaction (Figure 5), where endurance-trained lizards had lower maximal OCR than controls after addition of both FCCP (*p* = 0.003) and pyruvate (*p* = 0.03), but addition of oligomycin did not lower OCR differently between the training treatments (*p* = 0.84). Basal OCR of muscle fibers also did not differ between training treatments (*p* = 0.48). There was no significant effect of sex on OCR (*p* = 0.09). Taken together, these results indicate that untrained lizards had higher maximal respiratory capacity than trained lizards demonstrated by a greater increase in OCR after the addition of FCCP, which collapses the proton gradient (Figure 5). Both trained and untrained lizards further increased OCR with the addition of pyruvate, but control lizards elevated theirs to a higher extent (Figure 5).

## 4 Discussion

### 4.1 Effects of PQQ supplementation and training

Trained lizards alone exhibited low SMRs and high mitochondrial copy numbers, while PQQ-supplemented lizards exhibited high SMRs and low mitochondrial copy numbers compared to controls. Training was the only treatment that showed a significant increase in endurance performance. Therefore, the higher SMRs in PQQ-supplemented lizards are likely not due to increased expression of PGC-1α and subsequent mitochondria biogenesis. Additionally, the cause of performance enhancement in trained lizards is still ambiguous from the results of this experiment.

The PQQ supplement has antioxidant potential in addition to increasing mitochondria biogenesis, and it is possible that a synergistic effect occurs as both aerobic exercise and PQQ work to alter cellular metabolism (Hwang & Willoughby, 2018; Jonscher et al., 2021). For example, exercise increases ROS, which causes skeletal muscle damage and fatigue, detrimental to recovery (Hwang & Willoughby, 2018). Increases in ROS can function as a cellular stress stimulus and trigger factors with redox-sensitive signaling systems (Webb et al., 2017). Intracellularly, ROS leakage activates PPAR-γ signaling which increases mitochondria biogenesis through the increased expression of mitochondrial genes such as cytochrome c oxidase and isocitrate dehydrogenase (Webb et al., 2017). PQQ is then left to serve as an antioxidant rather than a stimulant for mitochondrial biogenesis. Rats given a PQQ supplement showed lower oxygen uptake, likely due to the duplicative role of PQQ in elevated antioxidant defense against ROS and mediating metabolic function (Hwang & Willoughby, 2018). The synergistic effect and antioxidant defense could explain the unchanged endurance in PQQ supplemented lizards. However, the low mtDNA counts and the elevated SMRs in supplemented lizards, as well as enhanced endurance, despite low mtDNA copy counts in trained + supplemented lizards, requires more detailed investigation into the synergisticeffect of PQQ action and exercise on mitochondria respiratory capacity and function.

Although the antioxidant role of PQQ supplements could inhibit the increase in mitochondria biogenesis, PQQ did not hinder all exercise adaptations in our first study group as demonstrated by the increased endurance in lizards with training + PQQ supplement, as well as the lack of a training*PQQ interaction (Figure 1). That is, trained lizards had enhanced endurance whether they received PQQ or not. This indicates that there are further physiological adaptations outside of mitochondria biogenesis involved in increasing aerobic capacity. These results led to our second experiment on the role of mitochondria function and adaptation in enhanced endurance after aerobic exercise.

### 4.2 Mitochondria function—mito stress test

Trained lizards exhibited lower resting metabolic rates and lower maximal mitochondrial respiratory capacity than controls, indicating differing whole-organism and mitochondrial adaptations in response to higher energy demands from exercise training. Individual mitochondria can respond to demands by adjusting activity within the electron transport chain such as proton concentrations contributing to proton-motive force across the inner mitochondrial membrane, which affects ATP synthesis, proton leak, and ROS production (Treberg et al., 2018; Koch et al., 2021; Mendez-Romero et al., 2022). When oxygen consumption is uncoupled from ATP synthesis, individual mitochondria found within different organisms can have similar OCR with very different ATP synthesis rates (Koch et al., 2021). Very little is known about these details in reptiles, so below we speculate about what happened in our system with the goal of providing testable hypotheses for future research.

Mitochondria of endurance-trained lizards and control lizards are likely prioritizing different efficiencies. Koch et al., 2021 proposed different energetic strategies that balance ATP synthesis and ROS production. An organism that prioritizes higher mitochondria efficiency will be effective in making ATP when demand is high by producing more ATP per oxygen molecule consumed. However, higher efficiency mitochondria will make higher levels of ROS when at rest or when ATP demand is low. Future studies should quantify antioxidant levels in trained lizards to see if there is a compensatory effect to ameliorate this possibility. The low mitochondria efficiency strategy allows for the organism to still meet ATP demands while limiting ROS production at rest and sacrificing more overall proton leak. Green anole lizards rely primarily on burst anaerobic locomotion when escaping predators and catching prey, though not for foraging and daily movements in their home ranges (Jenssen et al., 1995; Irschick & Losos, 1998; Irschick et al., 2005), and the low-efficiency strategy may be advantageous for that lifestyle. Our training, with chronic elevation of aerobic locomotion, appears to have pushed them to a high-efficiency strategy. It is also possible that the more sedentary time in captivity pushed our control lizards to an even less efficient strategy than that observed in nature.

The high-efficiency adaptation explains why less oxygen is consumed by trained lizards than untrained lizards at the whole-organism level (RMR and SMR) and after the addition of FCCP and pyruvate. It is possible that endurance-trained lizards (with higher mtDNA copy number; see also Liu et al., 2017), which often experienced high energy demands, prioritized higher mitochondria efficiency to increase ATP production per oxygen molecule consumed. Untrained lizards did not experience high energy demand during the experiments, therefore, a lower mitochondria efficiency allows high energy demands to still be met by consuming more oxygen for ATP synthesis (with lower mtDNA copy numbers), while maintaining low levels of ROS during a more constant state of low energy demand. Trained lizards with high efficiency would have low proton-motive force and proton leak as a high demand for energy causes them to quickly use up the proton gradient; untrained lizards with low efficiency would likely experience higher levels of proton leak. Although we were unable to sufficiently analyze proton leak, these efficiency adaptations seem to explain the dramatic difference in maximal respiration. Nevertheless, we did not detect differences in ATP-linked respiration (indicated by a difference in OCR after addition of oligomycin), so further studies are needed to test this hypothesis.

Although our second study group did not include PQQ supplementation, it is possible they would also be pushed to lower mitochondria efficiency. With low endurance performance, high SMRs, and low mtDNA copy numbers, we would expect supplemented lizards to exhibit higher maximal respiratory capacities due to higher oxygen consumption rates needed to meet high energy demands. However, it is possible that the combination of training and supplementation could allow for a more optimal, higher mitochondria efficiency due to the synergistic antioxidant effect of PQQ, allowing for more efficient ATP synthesis during high energy demand while limiting damaging ROS during low energy demand.

We also note that resistance training and aerobic exercise can have mitochondrial adaptations not measured here, which may have been present in our trained lizards. High energy demands can increase inter-mitochondrial junctions and density of inner mitochondrial membranes, presumably coordinating cristae within mitochondria as an adaptation to increase performance in response to a stressor (Heine & Hood, 2020). Chronic exercise has been shown to increase mitochondria fusion in adult human skeletal muscle as well (Arribat et al., 2019), which indicates the importance of an increase in mitochondrial content and turnover to meet the oxidative demands of exercise and maintain mitochondrial function. Alternatively, a potentially unique mechanism seen in extremely small mammals could explain how lizards could maintain increased efficiency. Small mice consume less oxygen than larger species by increasing the amount of ATP produced per molecule of oxygen via better coupling efficiency within mitochondria (Böel et al., 2020). Better coupling efficiency and less proton leak may allow these small mice to rely on muscle activity for thermoregulation rather than proton leak when ATP demand is high (Böel et al., 2020), and the higher muscle activity could be sustained with the higher mitochondrial efficiency (Böel et al., 2020). Lizards, as ectotherms, do not need to rely on proton leak to generate heat for thermoregulation, which could allow them to improve coupling efficiency in mitochondria without the cost of lost heat production that is seen in mice. Future studies will help to determine whether these mitochondrial responses are found in lizards.

## 5 Conclusion

Aerobic exercise increased endurance performance and decreased whole-organism metabolism in both of our experiments. We concluded that increased aerobic performance was likely not due to mitochondria biogenesis alone when PQQ supplemented lizards did not increase endurance performance and exhibited a decrease in mtDNA copy numbers, while trained lizards exhibited an increase in mtDNA copy numbers but decreased SMRs. The second experiment addressed mitochondria function using a Mito Stress Test and demonstrated altered mitochondria function after aerobic exercise, with endurance trained lizards meeting ATP demands with lower OCR, and control lizards reaching higher maximal respiration. Our results suggest that endurance-trained lizards altered mitochondria function by shifting to a higher efficiency, which includes the ability to meet high ATP demands without consuming more oxygen and likely having less proton leak but higher levels of ROS when at rest. Control lizards likely maintained a lower mitochondria efficiency, which allowed them to meet ATP demands while minimizing ROS production and sacrificing higher proton leak. It is possible that the addition of PQQ supplement and its antioxidant defense would allow for a higher mitochondria efficiency adaptation without high levels of ROS when at rest. However, we were limited in our interpretation by not including PQQ in the second experiment and not being able to measure all mitochondrial adaptations. Future studies that look specifically at ROS and proton leak, as well as antioxidant production, could help determine the most important role of mitochondria performance and functional adaptations in aerobic performance of lizards.

## Data Availability

The raw data supporting the conclusion of this article will be made available by the authors, without undue reservation.
