# Supplementary material for: How does mitochondria function contribute to aerobic performance enhancement in lizards?
Source: Front Physiol. 2023 May 5;14:1165313. doi: 10.3389/fphys.2023.1165313 (PMC10198381; doi:10.3389/fphys.2023.1165313)
Supplement: Supplementary file 1 [file Table1.docx]

**Table S1.** Primers used in quantitative PCR to determine mitochondrial gene copy number.

| mtA F—CCACATGACAAAAAATTGCCCCC  R—CTGACGTGGATAAGTTGTTTA |
| --- |
| mtB F—CTAAACCAAGAGCCTTCAAAGCTC  R—CGAATAGAAGCCCGCTGG |
| HSD17 F—AGACATTTGTACCACGGTCC  R—CAGTTTCCTCCCTCCATAATGG |
